# Supplementary figures and images for: Impact of examined lymph node count on long-term survival of T1-2N0M0 double primary NSCLC patients after surgery: a SEER study
Source: PeerJ. 2020 Feb 26;8:e8692. doi: 10.7717/peerj.8692 (PMC7049255; doi:10.7717/peerj.8692)

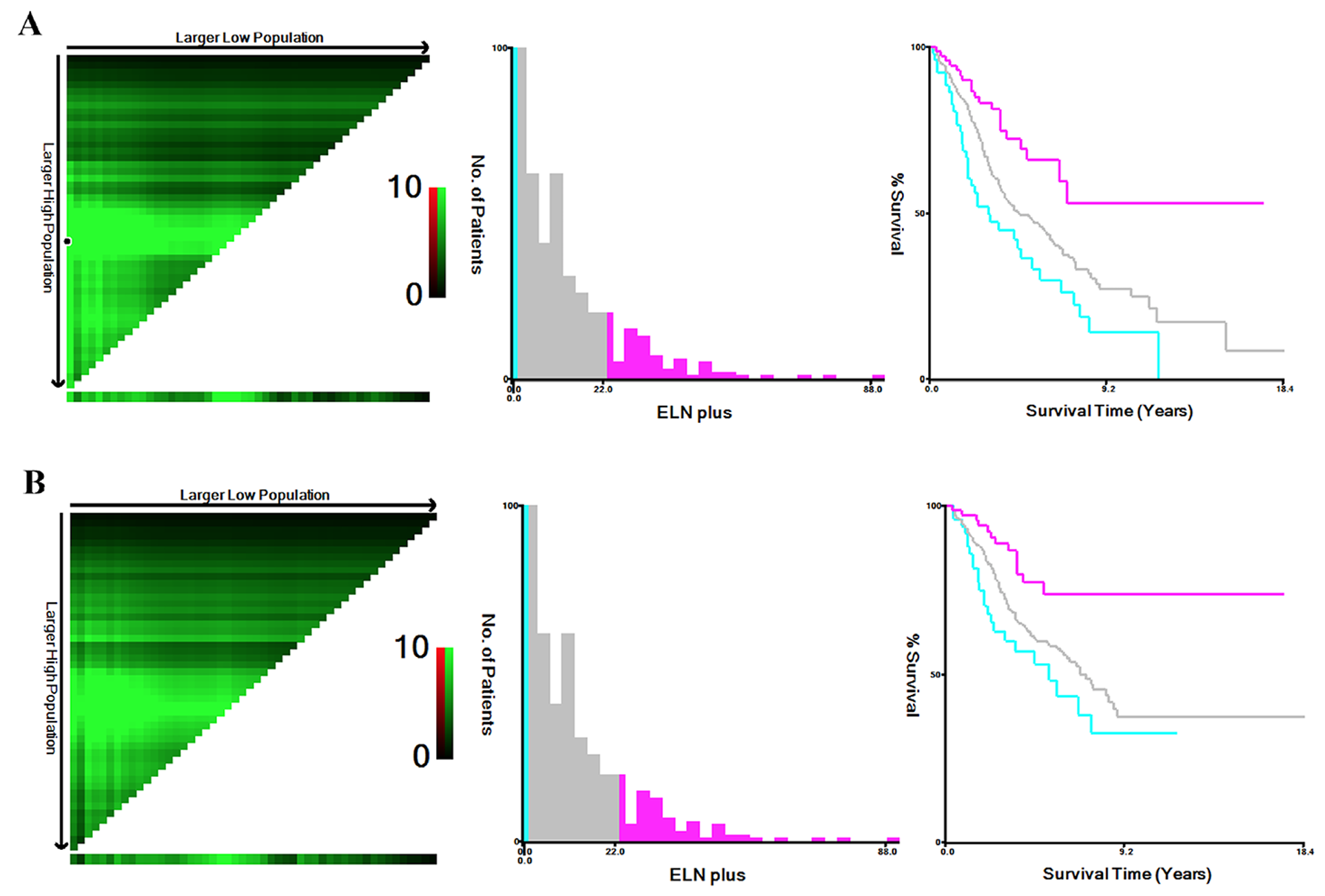

Supplement: Supplemental Information 1 — X-tile plots of the training sets are shown in the left panel, with plots of matched validation sets shown in the smaller inset. The optimal cutoff point is shown on a histogram of the entire cohort (middle panels), and a Kaplan–Meier plot (right panels). [file peerj-08-8692-s001.png]

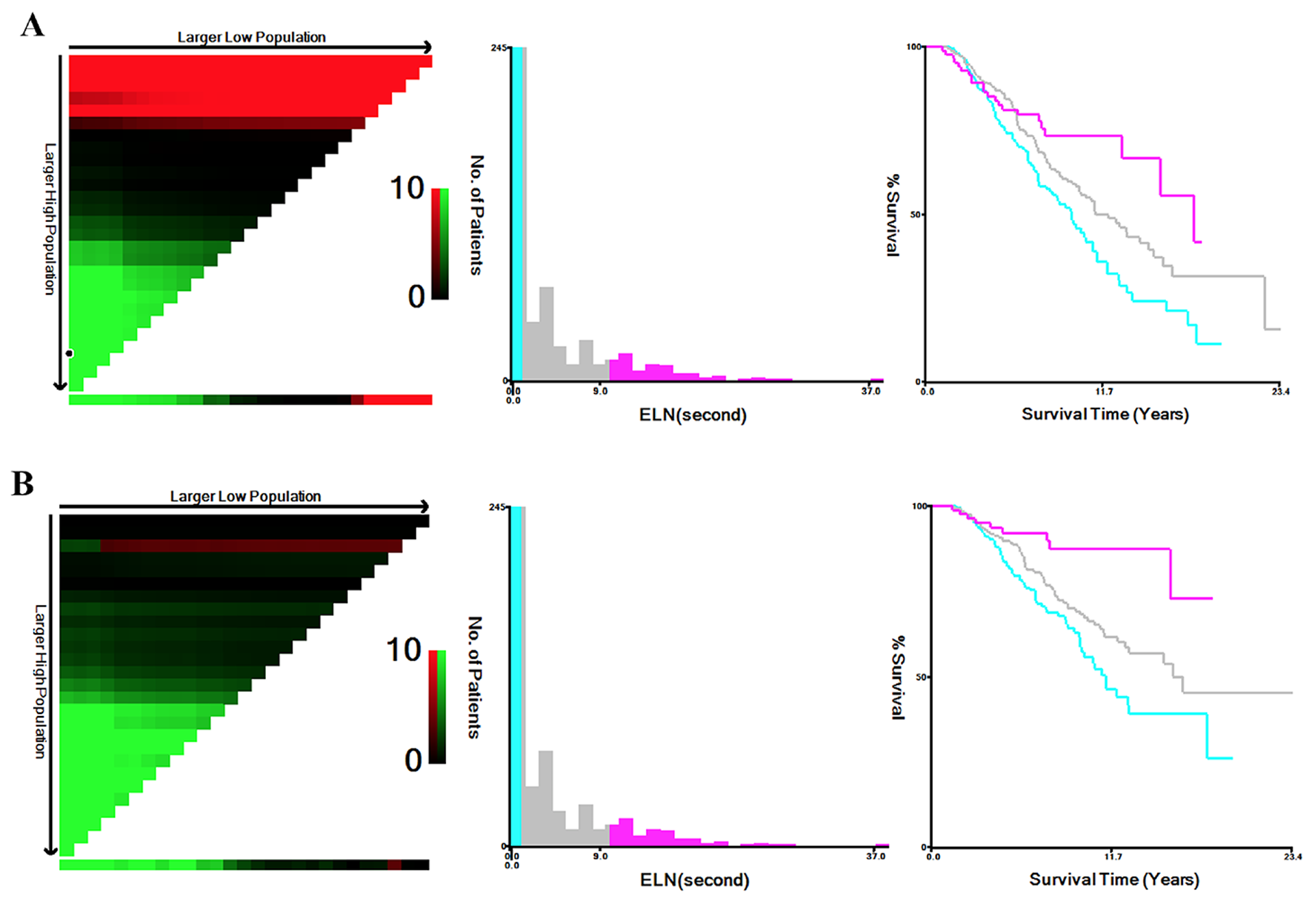

Supplement: Supplemental Information 2 — X-tile plots of the training sets are shown in the left panel, with plots of matched validation sets shown in the smaller inset. The optimal cutoff point is shown on a histogram of the entire cohort (middle panels), and a Kaplan–Meier plot (right panels). [file peerj-08-8692-s002.png]
