# Supplementary material for: Impact of examined lymph node count on long-term survival of T1-2N0M0 double primary NSCLC patients after surgery: a SEER study
Source: PeerJ. 2020 Feb 26;8:e8692. doi: 10.7717/peerj.8692 (PMC7049255; doi:10.7717/peerj.8692)
Supplement: Supplemental Information 3 [file peerj-08-8692-s003.docx]

**Supplement 3.** Comparison between patients with different ELN plus in SDP-NSCLC group by multivariate Cox regression

| **ELN plus** | **Overall Survival** | | | **Cancer-specific Survival** | | |
| --- | --- | --- | --- | --- | --- | --- |
|  | **HR** | **95%CI** | ***P* value** | **HR** | **95%CI** | ***P* value** |
| 0 vs. 1-22 | 0.641 | 0.445-0.923 | **0.017** | 0.631 | 0.402-0.992 | **0.046** |
| 0 vs.＞22 | 0.304 | 0.176-0.527 | **0.001** | 0.284 | 0.143-0.561 | **0.001** |
| 1-22 vs.＞22 | 0.475 | 0.297-0.760 | **0.002** | 0.449 | 0.252-0.800 | **0.007** |

Notes.

SDP-NSCLC, synchronous double primary non-small cell lung cancer; HR, hazard ratio; CI, confidence interval; ELN, examined lymph node.
