# Supplementary material for: Impact of examined lymph node count on long-term survival of T1-2N0M0 double primary NSCLC patients after surgery: a SEER study
Source: PeerJ. 2020 Feb 26;8:e8692. doi: 10.7717/peerj.8692 (PMC7049255; doi:10.7717/peerj.8692)
Supplement: Supplemental Information 4 [file peerj-08-8692-s004.docx]

**Supplement 4.** Comparison between patients with different ELN 2 in MDP-NSCLC group by multivariate Cox regression

| **ELN 2** | **Overall Survival** | | | **Cancer-specific Survival** | | |
| --- | --- | --- | --- | --- | --- | --- |
|  | **HR** | **95%CI** | ***P* value** | **HR** | **95%CI** | ***P* value** |
| 0 vs. 1-9 | 0.676 | 0.504-0.906 | **0.009** | 0.689 | 0.488-0.973 | **0.035** |
| 0 vs.＞9 | 0.411 | 0.253-0.668 | **＜0.001** | 0.223 | 0.109-0.459 | **＜0.001** |
| 1-9 vs.＞9 | 0.608 | 0.376-0.985 | **0.043** | 0.324 | 0.158-0.664 | **0.002** |

Notes.

MDP-NSCLC, metachronous double primary non-small cell lung cancer; HR, hazard ratio; CI, confidence interval; ELN, examined lymph node
