# Supplementary material for: Impact of examined lymph node count on long-term survival of T1-2N0M0 double primary NSCLC patients after surgery: a SEER study
Source: PeerJ. 2020 Feb 26;8:e8692. doi: 10.7717/peerj.8692 (PMC7049255; doi:10.7717/peerj.8692)
Supplement: Supplemental Information 6 [file peerj-08-8692-s006.docx]

Race: 1:white; 2:black; 3:other

Sex: 1:male; 2:female

Location: 1:Ipsilateral; 2:Bilateral

Interval time: month

Group: 1:synchronous; 2:metachronous

**First tumor**

Primary site: 1:Upper lobe; 2:Middle lobe; 3:Lower lobe; 4:Overlapping lesion

Grade: 1:Ⅰ; 2:Ⅱ; 3:Ⅲ; 4:Ⅳ

Laterality: 1:Left; 2:Right

Size: millimeter

**Second tumor**

Primary site: 1:Upper lobe; 2:Middle lobe; 3:Lower lobe; 4:Overlapping lesion

Grade: 1:Ⅰ; 2:Ⅱ; 3:Ⅲ; 4:Ⅳ

Laterality: 1:Left; 2:Right

Size: millimeter

OS status: 1:dead; 0:alive

CSS status: 1:dead; 0:alive

Survival time: month
